# Nasal Cytology as a Marker of Atopy in Children

**DOI:** 10.1155/2017/4159251

**Published:** 2017-09-05

**Authors:** Anna Mierzejewska, Anna Jung, Bolesław Kalicki

**Affiliations:** Department of Pediatrics, Pediatric Nephrology and Allergology, Central Clinical Hospital of the Ministry of Defence, Military Institute of Medicine, Warsaw, Poland

## Abstract

The aim of this study was to evaluate the cytological picture of nasal mucosa in children with atopic diseases and to determine the diagnostic value of the test for the diagnosis of atopic diseases. The study included 140 children from 4 months to 17 years old. Among children with a history of atopy, there were 30 children with atopic dermatitis, 30 children with asthma, and 46 children with allergic rhinitis. The control group consisted of 34 healthy children. The nasal scraping technique has been used to collect samples from the nasal cavity. The samples were evaluated under light microscope. Epithelial cells as well as infiltrating cells were assessed. The only statistically significant group of cells differentiating children with atopic disease and without atopy were eosinophils, which in children with atopy were significantly more common. Assuming a significant eosinophilia value of at least 5% of all cells in cytogram, the sensitivity of nasal cytology in allergic rhinitis was 52.2%, in asthma 33.3%, and in atopic dermatitis 13.3%. The specificity of the test in atopic diseases was 94.1%. It can be concluded that nasal cytology with eosinophilia assessment can be a useful tool for an early diagnosis of atopic disease in children.

## 1. Introduction

The nasal mucosa is the most accessible for the noninvasive study part of the respiratory system. The nasal cytology is a research method evaluating cells located within it, and it is mainly used as an additional test in otolaryngology. Epithelial cells such as basal cells, ciliated and nonciliated columnar cells, mucous (goblet) cells, and squamous cells are assessed as well as infiltrating cells: neutrophils, eosinophils, mast cells, basophils, lymphocytes, and monocytes.

This method allows to assess the pathophysiological changes occurring in the nasal mucosa and monitor response to applied treatment and thus has both diagnostic and therapeutic values. It becomes especially valuable in monitoring changes in the nasal mucosa of a particular patient [1–3]. An extremely precious feature of this research, especially in the pediatric population, is its noninvasiveness and painlessness; moreover, it requires no anesthesia, and it is cheap and simple to make.

The nasal cytology detects changes in proportions of the epithelial cells in response to external factors and infectious agents of various etiology [4–7]. Most often, this test is used in otolaryngology for the differentiation of allergic, nonallergic, and infectious rhinitis [8, 9].

This method has also been assessed as useful in the case of nonallergic diseases in children—chronic sinusitis and nasal septum deviation, as well as latent chronic sinusitis in children with asthma [10, 11]. Nasal cytology is also used in adult patients with nasal polyps [12, 13].

A microscopic evaluation of obtained slides relies on the confirmation of the presence of particular types of cells in the fields. Currently, no standardized analytic method exists for doing cell counts, and therefore, it is difficult to compare studies conducted in different centers [2]. Moreover, it is hard to establish clear criteria between pathology and physiology of cytological nasal mucosa image. Such assessment is based on the superiority of one group of cells over the other. In normal cytogram columnar cells, particularly ciliated, predominate while neutrophils and basal cells are found only as single cells in the slide. Eosinophils and mast cells in the nasal mucosa of healthy individuals do not occur [2, 8].

The pathophysiological processes, occurring in the nasal cavity, first appear in the ciliated cells. This leads to the transformation of the cells, depletion of ciliated columnar cells in favor of mucus-secreting goblet cells [8]. Both chronic stimulation of the respiratory irritants and sudden external stimuli may increase the intensity of goblet cells. Furthermore, there is an increase in goblet cell size and intracellular amounts of mucin. In turn, this leads to increased mucus production and impaired ciliary mucus transport. Accumulating amount of secretions in turn increases the risk of bacterial infection [8].

The presence of migrant cells also provides valuable information about the pathophysiological processes occurring in nasal mucosa scrapings.

Atopy is defined as the intersubject and/or family genetically determined tendency to the production of specific IgE antibodies and the occurrence of hypersensitivity in response to environmental antigens (allergens) [14]. This feature is revealed in the form of atopic diseases, which include atopic dermatitis, allergic rhinitis, and asthma.

High prevalence of atopic diseases and its increased incidence over the last decades of about 20% of the population makes them important clinically and an epidemiological problem [15].

## 2. Aim of the Study

The aim of this study was to evaluate the cytological picture of nasal mucosa in children with atopic diseases. In addition, the objective was also to determine the suitability of the nasal mucosa for the diagnosis of atopic disease, taking into account the sensitivity and specificity of the test.

## 3. Materials and Methods

The study enrolled a total of 140 children including 76 boys (54.29%) and 64 girls (45.71%). The youngest examined child was at the age of four months, the oldest was 17 years old, and the average age was 9 years and 5 months.

All children enrolled in the study were patients of the Department of Pediatrics, Pediatric Nephrology and Allergy, Military Institute of Medicine, Warsaw. Group I included children up to the age of 18 remaining under the allergology care with the diagnosis of atopic disease: atopic dermatitis (AD), asthma, or allergic rhinitis (AR). Among children with a history of atopy, there were 30 children with AD, 30 children with asthma, and 46 children with AR. Group I consisted of a total of 106 children, including 61 boys and 45 girls. Due to the possibility of coexisting atopic diseases, in order to ensure better reliability of the results, only children diagnosed with atopic disease were eligible.

The diagnosis was based on the Hanifin and Rajka criteria for atopic dermatitis, criteria consistent with the ARIA for allergic rhinitis and GINA for asthma.

Exclusion criteria for patients in group I participating in the study were current respiratory tract infection, usage of nasal corticosteroids within 14 days preceding the survey, usage of antihistamines within 14 days preceding the survey, and the lack of consent of the caregiver and/or patient.

Group II (control group) consisted of children without any features of atopic disease both in pediatric examination and the interview following the nasal sampling. Among the control group, there were 34 children, including 15 boys and 19 girls, hospitalized at the clinic for reasons unrelated to atopic diseases. The average age was 12 years and 4 months. Exclusion criteria for patients classified to group II included current respiratory tract infection, atopic disease in the form of atopic dermatitis, allergic rhinitis, or asthma at any time in life, and the lack of consent of the caregiver and/or patient.

Each test was preceded by obtaining informed consent from the parent, and in the case of a child over 16 years of age, also his consent is obtained. In the case of the examination in the nasal cavity of the secretions, the sample was taken after purging the nose. In each patient, the material for cytological examination has been collected from the middle third of the inferior turbinate. The samples were taken from one nasal cavity with nasal scraping method (using a nasal curette) and then transferred to a microscopic slide.

After the cytologic smear has been prepared, it has been fixed using “Cytofix” and then stained with hematoxylin and eosin. The slide was evaluated under a light of Delta Optical Evolution 300 microscope in a 400x magnification. The analysis was based on identifying in successive fields the presence of particular cell types: ciliated and nonciliated columnar cells, mucous (goblet) cells, basal, and squamous cells, neutrophils and eosinophils. The method was semiquantitative in nature, and at least several hundred cells in representative fields of view had been counted. The resulting cytogram presented the percentage of individual cell types in the preparation.

No adverse effects were observed in any child which could result from the research itself.

The study was approved by the Bioethics Committee of the Military Institute of Medicine. The analysis was performed using Statistica version 12. Statistical differences were considered statistically significant for which the level of statistical significance fulfilled the condition of *p* < 0.05. The significant eosinophilia of 5% has been adopted for the evaluation of the sensitivity and specificity of the test. The sensitivity of the study was defined as the ratio of true positive results to the sum of the true positive results and false negative results. The specificity of the study was defined as the ratio of truly negative results to the sum of truly negative and false positive results.

## 4. Results

As shown in the table above, eosinophils are the only statistically significant group of cells differentiating children with atopic disease and without atopic disease—there are significantly more eosinophils in the atopic patients (*p* = 0.02) (Table 1).

**Figure 1:**
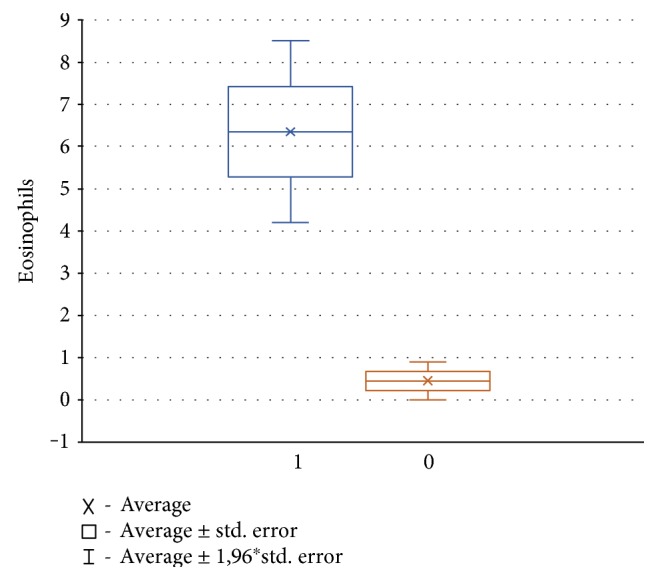
The median with spread interest projection of eosinophils in children with atopic disease and the control group (1—atopic disease, 0—control group).

Mean number of eosinophils in nasal smears, in children with atopy, was significantly higher in comparison to the group of children without atopic disease (Figure 1).

In order to assess diagnostic value of nasal mucosa, the sensitivity and specificity of the test were calculated. The obtained results are presented in Table 2.

## 5. Discussion

The diagnosis of allergy in children, especially in the youngest age groups, is a difficult challenge and requires careful and broad analysis. This is due to both the diversity of the symptoms of the disease and absence of specific and reliable laboratory tests.

Despite the fact that nasal cytology is a simple, noninvasive, repetitive, and cheap method, its use in medicine is relatively rare.

Cytological examination of nasal mucosa has a long history. Most of the papers had paid attention to the presence of eosinophils and their relation to the allergy, and the first of them had been conducted more than a century ago by Gollash in patients with asthma [16]. Then, in the twentieth century, Eyermann has proven the presence of eosinophils in 72% out of 92 patients with rhinitis [17]. Fink's studies evaluated the cellular changes in the nasal mucosa in various conditions and indicated the presence of eosinophils in anaphylaxis [18]. During this period, Hansel published the results of analyzes of nasal mucosa and paranasal sinuses in more than 1000 patients, in which he emphasized the role of eosinophil in the diagnosis of allergic diseases [19]. Nasal eosinophilia was evaluated in subsequent years by Bryan WTK and Bryan MP, Murray et al., and Mygind [20–22].

Malmberg and Holopainen demonstrated the correlation between nasal eosinophilia and allergic rhinitis [23].

The studies of nasal cytology in children were initiated in the 50s of the twentieth century by Matheson et al. and indicated the presence of eosinophils in up to 30% of infants [24]. However, later studies conducted by Cohen et al. did not confirm that thesis [25].

Relationship between atopy and nasal eosinophilia has been also studied by Kajosaari and Saarinen. In this survey, a group of 178 children with atopic diseases was observed from 3 years of age; the presence of eosinophils and mast cells in cytograms obtained from the nasal mucosa (using nasal smear taken by wiping the mucosa with a cotton-tipped applicator) was an important indicator of atopy, characterized by a high specificity but a low sensitivity [26].

Further studies, conducted with more accurate scraping technique, have provided new information. In the study of Tarchalska-Kryńska on a group of 105 newborns, an absence of goblet cells had been observed. Moreover, there were two dominating types of cytograms: with a predominance of at least 50% of the columnar cells (36.19% in children) and with a predominance of at least 50% of neutrophils (in 41.9% of children), while eosinophils were found in 7.6% of cases [27]. Over the last years, studies on a large group of the population, including children with various forms of chronic rhinitis, have also been led by Gelardi et al. [4, 7].

Zeiger and Heller's prospective studies, carried out in the 90s of the twentieth century, have shown an increase in eosinophils and basophils in children with a family history of allergic diseases followed from birth to 4 years of age. These changes in cytograms have not been reported in children without allergic diseases [28].

In a similar study, Borres analyzed cytograms of infants with a positive family history of allergies and then repeated the study in 18 months of age. In children with symptoms of atopy (this group included both children with symptoms of respiratory disease and the symptoms of AD), metachromatic cells (mast cells and basophils) were more common compared to the patients without symptoms of atopy. Eosinophils were in turn found in both groups of children without significant correlation [29].

Nowacki et al. analyzed the usefulness of cytology of the nasal mucosa in predicting the occurrence of atopic diseases (atopic dermatitis, asthma, and allergic rhinitis) in children up to 4 years of age. It has been shown that eosinophilia in nasal cytology, of at least 8%, was associated with a high risk of developing AR. Therefore, it has been proposed that the increased nasal eosinophilia in young children can be taken as an indicator of the risk of allergic march. The average percentage of eosinophils at baseline was significantly higher in children who were finally diagnosed with allergic rhinitis in relation to children diagnosed with atopic dermatitis (18% versus 3%, *p* < 0.004), or diagnosed with nonallergic disease (18% versus 3%, *p* < 0.001) [30].

In the study conducted by DeMuth et al., children aged 2–47 months suffering from atopic dermatitis had significantly more eosinophils in cytology of the nasal mucosa compared to control group [31].

The present study covered a total of 140 children, out of which 34 constituted the control group. In the group of children with atopic disease, eosinophils were observed significantly frequent (*p* = 0.02). Based on the results obtained, it can be assumed that nasal eosinophilia is a good marker of atopy in children.

The present study has determined the sensitivity and specificity for the study for atopic dermatitis, allergic rhinitis, and asthma, depending on the degree of eosinophilia. As a positive test, the presence of eosinophils in at least 5% of all cells was considered positive.

Evaluation of the sensitivity and specificity in the presented study has been based on the presence of eosinophils in the cytological picture, assuming eosinophil counts of 5% of the cells in the cytogram. By analyzing individual studies evaluating the problem of eosinophilia in the cytological picture, one can see the differences in the very definition of the term “significant eosinophilia.” This range is wide and it is ranging from 4% of all cells in Miller et al. [32, 33], through 10% in Mygind [22, 34], and to as much as 25% in Burrows et al.'s work. On the other hand, Jankowski et al. adopted a cut-off value of 20% [35], and Twarduś and Lis—10% [36]. Vaidya et al. found eosinophilia in at least 5% of these cells in the cytogram [37].

According to other researchers, the presence of eosinophils in the cytology of the nasal mucosa can be attributed to the pathology itself [38]. Although in the first studies in children, Matheson et al. have described the presence of eosinophils in as many as 30% of newborns [24], and further studies have not confirmed this. In Cohen et al.'s study among 22 healthy infants, no eosinophil was found in the cytological analysis of any of the children [25].

In Tupieka-Kołodziejska's work, in a group of 337 newborns (both as full term and premature) in more than 95% of cases, there were no eosinophils identified; whereas in children aged 12–18 months, eosinophils occurred in 11.9% of children [39].

In the semiquantitative assessment in Meltzer et al.'s classification, already 1+ (average number of cells with 10 fields of view in 1000x magnification equals 1.1–5.0) means eosinophilia [40].

For eosinophilia at 5%, the sensitivity of the study in AR was 52%, only 33% for asthma, and 13% for AD; at the same time, the specificity amounted to 94%.

A similar analysis was made by Miller et al., however assuming 4% as a criterion for eosinophilia. In their work, the sensitivity of the study in allergic rhinitis was 70%, with a specificity of 94% [32]. In the Bakhshaee et al.'s study, eosinophilia diagnosis was assessed as a highly specific (88.5%) but less sensitive (51.3%) in the cytological examination of the nasal mucosa in patients with AR, with eosinophil counts of at least 10% number of cells in the smear [41]. For eosinophilia, defined as the presence of at least 20% cells, Lans et al. have demonstrated a sensitivity of 43%, a specificity of 98%, a positive predictive value of 96%, and a negative predictive value of 56% [42].

In a study by Miri et al., the sensitivity of a study conducted on a group of over 4000 children with AR was at 62% and specificity was at 96% [43]. The results obtained by other researchers in the group of children with allergic rhinitis seem to coincide with the presented work. They exhibit moderate sensitivity of the test at high specificity of nasal cytology.

The incidence of eosinophilia in nasal swabs in children with asthma was assessed in Nagayama et al.'s work, and it was 21%, 64%, and 75–78%, respectively, for children under 1 year of age, 1 year old, and 2-3 years of age. Eosinophilia in the above-mentioned publication recognized the presence of at least 11 cells per 5 fields of view at a 1000-fold magnification [44].

On the other hand in the Kumar et al.'s study, eosinophilia (i.e., eosinophil counts of at least 10%) was observed in 52.4% of children with AR and in 64.6% of children with asthma and AR [45]. In Prabhu et al.'s study conducted among adults, at least 5% of nasopharyngeal eosinophils were identified among 44% of patients with asthma and 49% of patients with asthma and AR [46]. Shaheen et al. also have studied the presence of eosinophils in a cytological study in asthmatic children with significant differences in the control group. In this study, eosinophils were found in 20%, 53%, and 27% of patients with severe, moderate, and mild asthma, respectively [47].

In a presented study, the presence of eosinophilia at a level of at least 5% was found in 33.3% of children with asthma. This is a lower percentage than in the cited studies. However, it is worth emphasizing that for the safety of children, chronic inhaled glucocorticosteroids in children were not being paused before sampling. In addition, the lower eosinophilia value in asthmatic children obtained from self-examination may be due to the fact that patients did not have coexisting allergic rhinitis, as was the case in other cited cases.

Based on the results of the study, the presence of eosinophil in nasal cytology is a good indicator of the likelihood of allergic rhinitis and in combination with other diagnostic tests leading to proper diagnosis. In the case of asthma and atopic dermatitis, its sensitivity is already considerably lower and insufficient to recommend this test for the diagnosis of the above conditions. At the same time, considering the possibility of allergic march in children diagnosed with AD/asthma, it seems to be helpful in the early detection of allergic rhinitis in these groups of children.

It should be noted that both, the sensitivity and specificity of the test, depend on the proper sampling and preparation of the slides as well as the skillfulness of the examiner. In the study of Turkish researchers, there were differences in the cytograms assessed by two different researchers—these differences were related to the amount of neutrophils, basophils, and the total number of inflammatory cells. Nevertheless, it did not concern the amount of eosinophils [48]. An additional difficulty to obtain a reliable test result is that the eosinophils can be present in the preparation irregularly forming groups. It is worth emphasizing that until now, there have not been established any standards for testing and analysis of samples and that there is no consensus defining the value of “significant eosinophilia” in the nasal cytology.

## 6. Conclusions

Nasal cytology with eosinophilia assessment can be a useful tool for early diagnosis of atopic disease in children. Nasal cytology in children with allergic rhinitis is a helpful diagnostic test with high specificity and moderate sensitivity. In children with atopic dermatitis and/or asthma, usefulness of nasal cytology is limited due to the low sensitivity of the method.

## Figures and Tables

**Table 1:** The results of the comparative analysis in the occurrence of individual cells between the group of children with atopic disease (1) and the control group (2).

	*t*-tests						
	Average group 1	Average group 2	*p* value	*N* group 1	*N* group 2	Standard deviation group 1	Standard deviation group 2
Eosinophils	6,30	0,41	0,002	106	34	10,89	1,23
Neutrophils	27,32	35,47	0,16	106	34	27,08	35,06
Ciliated/striate cells	49,32	50,41	0,84	106	34	26,42	29,71
Basal/squamous cells	9,48	6,97	0,36	106	34	14,42	12,57
Goblet cells	8,31	6,73	0,43	106	34	10,91	6,48

*p* values: significance of differences between subgroups of patients. *N*: number of patients investigated.

**Table 2:** The sensitivity and specificity of the test in children with atopic diseases.

	Sensitivity (%)	Specificity (%)
Allergic rhinitis	52,2	94,1
Asthma	33,3	94,1
Atopic dermatitis	13,3	94,1
